# Prediction of Soil Organic Carbon in a New Target Area by Near-Infrared Spectroscopy: Comparison of the Effects of Spiking in Different Scale Soil Spectral Libraries

**DOI:** 10.3390/s20164357

**Published:** 2020-08-05

**Authors:** Hongyang Li, Shengyao Jia, Zichun Le

**Affiliations:** 1 College of Computer Science and Technology, Zhejiang University of Technology, Hangzhou 310023, China; freelhy@126.com; 2 College of Mechanical and Electrical Engineering, China Jiliang University, Hangzhou 310018, China; 15a0106134@cjlu.edu.cn; 3 College of Science, Zhejiang University of Technology, Hangzhou 310023, China

**Keywords:** soil organic carbon, near-infrared spectroscopy, soil spectral library, spiking, partial least squares

## Abstract

Near-infrared (NIR) spectroscopy is widely used to predict soil organic carbon (SOC) because it is rapid and accurate under proper calibration. However, the prediction accuracy of the calibration model may be greatly reduced if the soil characteristics of some new target areas are different from the existing soil spectral library (SSL), which greatly limits the application potential of the technology. We attempted to solve the problem by building a large-scale SSL or using the spiking method. A total of 983 soil samples were collected from Zhejiang Province, and three SSLs were built according to geographic scope, representing the provincial, municipal, and district scales. The partial least squares (PLS) algorithm was applied to establish the calibration models based on the three SSLs, and the models were used to predict the SOC of two target areas in Zhejiang Province. The results show that the prediction accuracy of each model was relatively poor regardless of the scale of the SSL (residual predictive deviation (RPD) < 2.5). Then, the Kennard-Stone (KS) algorithm was applied to select 5 or 10 spiking samples from each target area. According to different SSLs and numbers of spiking samples, different spiked models were established by the PLS. The results show that the predictive ability of each model was improved by the spiking method, and the improvement effect was inversely proportional to the scale of the SSL. The spiked models built by combining the district scale SSL and a few spiking samples achieved good prediction of the SOC of two target areas (RPD = 2.72 and 3.13). Therefore, it is possible to accurately measure the SOC of new target areas by building a small-scale SSL with a few spiking samples.

## 1. Introduction

Soil is a natural resource that humans depend on for survival. Soil organic carbon (SOC) content is an important indicator to evaluate soil fertility, which directly reflects the soil quality and affects crop growth [1]. The measurement of SOC content is of great significance for accurate fertilization [2]. In addition, as a part of the global carbon cycle, studying the content and distribution of SOC helps human society understand the problem of global warming [3].

In recent decades, as a rapid, nondestructive, and accurate technique, near-infrared (NIR) spectroscopy has been widely used to predict soil nutrient information [4]. He et al. predicted soil macronutrient content using NIR spectroscopy, and showed that it is a very promising method for assessing nitrogen (N), organic matter (OM), and pH in soil [5]. Reda et al. successfully predicted SOC and total nitrogen (TN) content in four agricultural regions of Morocco using NIR spectroscopy [6]. However, in different geographic environments, the parent material, type, and usage of soil can be quite different, which means the existing calibration model can only be used to predict specific types of soil characteristics in certain areas. Therefore, if the existing calibration model was used to measure the soil characteristics of new target areas, the prediction accuracy may be greatly reduced, which greatly limits the application potential of the technology [7].

In order to improve the universality of the calibration model, some scholars suggested developing large-scale soil spectral libraries (SSLs) to ensure that the established model contained as many soil samples and types as possible [8]. Rossel and 45 other soil scientists developed a global visible–near-infrared (vis–NIR) SSL containing 23,631 soil samples, and showed that it can be used for both qualitative and quantitative analysis of soil [9]. Nocita et al. developed a European Union vis–NIR SSL with 19,969 topsoil samples within the framework of the Land Use/Cover Area frame Survey (LUCAS), and showed that a large-scale SSL can be used to predict SOC at continental scale with reasonable accuracy [10]. However, it requires much time, manpower, and financial resources to establish a global or national scale SSL containing many different types of soil samples. In addition, a unified spectral library depends on the same spectrometer measurement and spectral processing methods, making it difficult to establish a reliable large-scale library by sharing data.

Compared with the global and national scale, the provincial, city, and district scale SSLs are relatively easier to establish. In the past few years, 983 soil samples were collected across Zhejiang Province, and the NIR spectra and chemical SOC reference values of the soil samples were obtained. In this work, three SSLs of different scales were established: (1) a provincial soil spectral library (PSSL) containing 714 soil samples from Zhejiang Province, (2) a city soil spectral library (CSSL) containing 167 soil samples from Jinhua City, and (3) a district soil spectral library (DSSL) containing 102 soil samples from Wencheng District, Wenzhou City. The calibration models based on the three libraries were used to predict the SOC content of samples from two new target areas in Zhejiang Province. Therefore, the first objective of this paper was to compare and analyze the SOC predictive ability of the calibration models based on the three SSLs at different scales.

However, as the calibration model established by a small-scale SSL may be insufficient to predict a new target area, some scholars suggested adding soil samples from the target area to correct the calibration model by means of spiking [11]. The spiking method involves selecting representative soil samples from the target area, measuring their NIR spectra and chemical SOC reference values, adding the data of spiking samples to the calibration set, and then recalibrating the calibration model with the expanded dataset [12]. Guerrero et al. spiked models of different sizes with local samples of a target area and recalibrated them to predict the nitrogen content in soil samples, and the results showed that the small-scale model may be useful for local prediction after spiking [13]. Therefore, the second objective of this paper was to compare the prediction capability between unspiked models (UMs) and spiked models (SMs).

It was assumed that a recalibrated model after spiking would better adapt to the characteristics of soil samples in the target area, thereby improving the predictive ability of the model. However, the addition of spiking samples implies additional analytical testing, because the samples need to be analyzed for the chemical reference values [14]. If a small-scale SSL combined with a small number of spiking samples could achieve or even surpass the performance of a large-scale SSL, then the spiking method might be a more economical choice, because it could avoid the development of large libraries. Gogé et al. spiked national library models with 50 samples from target areas and significantly improved the models’ ability to estimate SOC [15]. Therefore, the third objective of this paper was to find a spiking strategy that would use as few spiking samples as possible to achieve an accurate prediction of new target area SOC based on a small-scale SSL.

This work explored how to use small-scale SSLs to predict the SOC of new target areas and studied the feasibility of the spiking method. It was expected that we would find an effective spiking strategy by adding different amounts of spiking samples and comparing the improvement effect of the predictive ability of the models, based on SSLs of different scales. In this work, three issues were mainly discussed and studied: (1) Does a larger SSL, on which the model is based, provide better prediction for new target areas? (2) Can the method of spiking improve the predictive ability of models built by SSLs of different scales? (3) What effect does the number of spiked samples have on the predictive ability of the model?

## 2. Materials and Methods

### 2.1. Soil Samples

The study area is located in Zhejiang Province (27°02′–31°11′ N, 118°01′–123°10′ E), People’s Republic of China. Zhejiang is located in the middle of the subtropical zone, which belongs to the subtropical monsoon climate. According to Chinese standard GB/T 17296–2009 (classification and codes for Chinese soil) [16], the soil of the province is classified as mainly yellow and red soil, mostly distributed in hilly and mountainous areas. In addition, paddy soil is distributed in the plain area, and some saline and desalinized soils are distributed along the coastal area.

First, 714 surface soil samples were collected from 11 prefecture-level cities in Zhejiang Province to form the PSSL. The soil types included red, yellow, paddy, and saline soil. At each collection point, five soil samples were collected from the surface layer (0–20 cm) according to the “plum blossom” sampling method [17]. After picking out the straw and stones, the five soil samples were mixed evenly as an experimental soil sample. In order to reduce the influence of particle size and moisture on the measurement results, each sample was sequentially processed by drying, grinding, and sieving (2 mm aperture). Each processed sample was divided into two parts: one part was sent to the Institute of Soil Fertilizer (Zhejiang Academy of Agricultural Sciences) to measure the chemical reference value of SOC content (g kg^−1^) by the Walkley-Black method [18], and the other part was stored in a Petri dish for NIR spectroscopy measurement and analysis. Then 167 CSSL soil samples were collected by the same method from Jinhua City (28°32′–29°41′ N, 119°14′–120°46′ E). The soil types in the CSSL were mainly red, yellow, and paddy soil. In addition, 102 DSSL soil samples were similarly collected from farmland in Wencheng District (27°34′–27°59′ N, 119°46′–120°15′ E), Wenzhou City. The soil type in the DSSL was mainly paddy soil.

**Figure 1:**
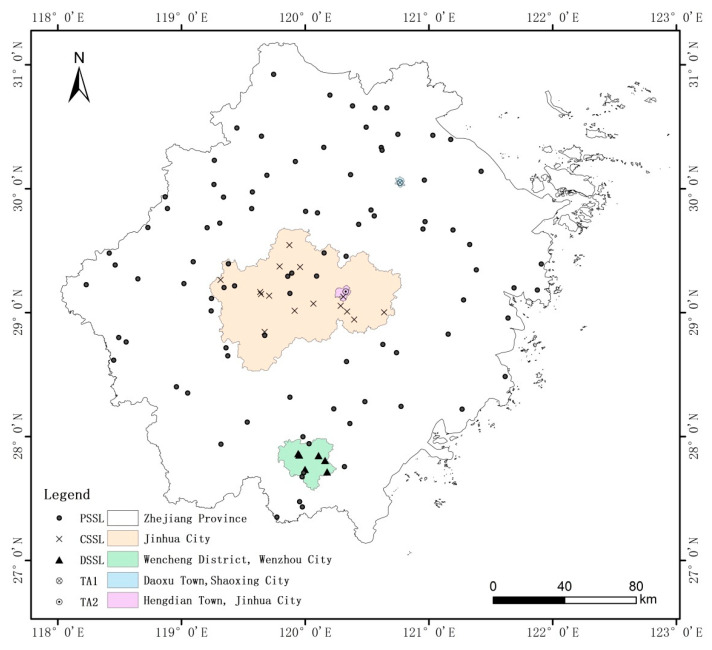
Map of three soil libraries and two target areas, and distribution of sampling points.

The first target area (TA1) was located in fields in Daoxu Town (30°00′–30°05′ N, 120°43′–120°48′ E), Shaoxing City. A total of 60 soil samples were collected. The typical soil type in TA1 was paddy soil, and the target fields in the area were mainly planted with double-season rice. The second target area (TA2) was located in fields in Hengdian Town (29°05′–29°13′ N, 120°14′–120°22′ E), Jinhua City. A total of 66 soil samples were collected. The typical soil types in TA2 were red, yellow, and paddy soil. The target fields in the area were mainly used for growing vegetables, soybeans, medicinal herbs, rice, corn, sweet potatoes, and other crops. A map showing the three soil libraries and two target areas, and the distribution of sampling points is shown in Figure 1.

In this work, the setting of the three soil libraries and the selection of the two target areas mainly considered two factors: first, the geographic scope covered by the soil libraries was from large to small, covering the provincial, municipal, and district scales in turn, and the corresponding soil sample quantity was also from large to small. In addition, as shown in Figure 1, the geographic scope of the PSSL included TA1 and TA2. The geographic scope of the CSSL included TA2, but not TA1. The geographic scope of the DSSL included neither TA1 nor TA2. Second, from the perspective of soil types, the PSSL contained all soil types in the province, but the proportion of paddy soil was not high (approximately 20%). Therefore, the target area with mainly the paddy soil type was selected as TA1 to study the universality of the large soil library. The soil types of the CSSL included both TA1 and TA2, but the number of soil samples was not as large as in the PSSL. The soil type of the DSSL was the same as TA1, but different from TA2. The main characteristics of all soil samples for the soil libraries and target areas are shown in Table 1.

### 2.2. NIR Spectral Data Measurement

In this work, a Bruker Fourier-type NIR spectrometer (Matrix-I, Bruker Optics Inc., Ettlingen, Germany) was used to measure the NIR spectral data of soil samples. The spectrometer was performed in the reflectance mode, the spectral measurement range of the instrument is 1000–2500 nm and the spectral resolution is 8 cm^−1^. Each spectrum contained 1555 wavelength variables. Further, each soil sample was evenly dispersed in the sample cup so that the thickness of the sample reached the height of the sample cup, which was 20 mm. The NIR light source penetrated the quartz window from bottom to top to illuminate the soil sample placed in the sample cup with a diameter of 50 mm. During the measurement, the soil sample rotated with the sample cup to obtain the average spectrum. The rotation speed of the sample cup was 4 cm/s, and each soil sample was scanned 64 times in 40 s. These scans were arithmetic averaged and the result used as the soil sample spectrum. The background spectrum of the spectrometer was obtained by the OPUS software when the sample cup was empty, and the background spectrum was recalibrated every 10 samples. All soil samples in this work followed the same procedure. The Matrix-I was directly connected to a computer via a network cable, and OPUS software (Bruker Optics Inc., Ettlingen, Germany) was used to control the spectrometer to collect data and process the data.

### 2.3. Modeling Methods

In the quantitative detection of SOC with NIR, the calibration set of representative soil samples should be selected first, and then the NIR spectrum and chemical SOC reference values of the soil samples in the calibration set are measured. In the NIR spectral data of soil samples obtained by the spectrometer, the information of various components was superimposed at each wavelength point. Due to the wide range of NIR spectral data and the serious overlap of spectral peaks, it is difficult to analyze directly. Therefore, it is necessary to extract effective information from the spectral data by chemometrics [19]. The calibration model between the NIR spectrum and the SOC reference values of soil samples was established by the chemometric method, so there was a certain statistical relationship between the spectrum and the corresponding SOC reference values. After establishing the calibration model, the NIR spectrum data of the new soil sample can be substituted into the model to predict the corresponding SOC.

The partial least squares (PLS) algorithm is a linear regression method commonly used in spectral data analysis [20]. The PLS algorithm simultaneously considers the spectral information and the corresponding physicochemical value information during modeling, and converts the original data into new variables termed latent variables (LVs), which are mutually orthogonal and uncorrelated through linear transformation. In this work, the optimal LVs used in the PLS model were determined by cross-validation with Unscrambler 9.7 software (CAMO Inc., Oslo, Norway).

**Figure 2:**
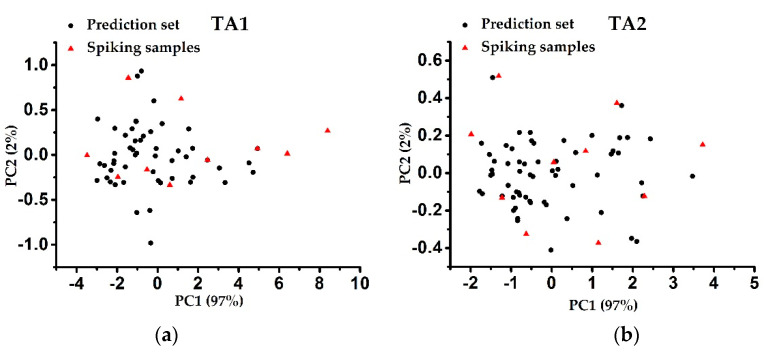
Score plots of (**a**) TA1 and (**b**) TA2 soil samples in principal component space defined by the first two principal components.

Three original calibration models without the target area samples added were established by the PLS method based on the data in the three SSLs, called the unspiked models (UMs). Then, in order to study the effect of spiking, the Kennard-Stone (KS) algorithm was applied to select the spiking subset from each target area [21]. The specific steps were as follows. First, principal component analysis (PCA) was performed on the NIR spectra of the samples in sets TA1 and TA2. Then, two principal components were generated in each case based on the predefined interpretation variance threshold (>99%). Second, five spiking samples were selected from the sample set of a target area by the KS algorithm, so that they were evenly distributed in the space defined by the first two principal components. Furthermore, in order to compare the effect of different numbers of spiking samples on the model, the KS algorithm was also used to select 10 spiking samples from the sample set in a target area. Score plots of the two target area samples in principal component space defined by the first two principal components are shown in Figure 2.

Finally, the remaining sample set of the target area was used as a prediction set to investigate the prediction capability of different models. Since for each target area at most 10 samples were selected for the original calibration model, the predicted sets of TA1_pre_ and TA2_pre_ consisted of 50 and 56 samples, respectively. The main characteristics of the two prediction sets are shown in Table 2.

### 2.4. Model Evaluation

In this work, the decision coefficient (R^2^_p_), root mean square error of prediction (RMSEP), bias, and residual predictive deviation (RPD) were used to evaluate the predictive ability of the model. R^2^_p_ was used to indicate the correlation between the predicted and the actual value of the model. The larger the R^2^_p_, the higher the correlation between predicted and actual value, and the better the model prediction effect [22]. The RMSEP was used to measure the deviation between predicted and the actual value. The smaller the RMSEP, the better the model prediction effect [23]. The RPD is an intuitive indicator used for evaluating a model’s predictive ability in soil spectroscopy. Specifically, when the RPD is less than 1.5, it indicates that the model effect is poor. When the RPD is 1.5–2.0, it indicates that the model effect is moderate, i.e., it can only distinguish physical and chemical values. When the RPD is 2.0–2.5, it indicates that the model effect is relatively good and it can be used for quantitative analysis. When the RPD is 2.5–3.0, it indicates that the model is very effective and can be used for quantitative analysis. When the RPD is greater than 3, it indicates that the model has a very good prediction effect [24].

### 2.5. Stastical Analysis

The RPD allowed us to compare the accuracy obtained in different prediction models. In order to analyze whether the difference between the RPD obtained based on different scales SSL is effective, the one-way analysis of variance (ANOVA) was performed using three scales of SSL as the single factor. The specific method is as follows: First, 70% of the soil samples in PSSL were randomly selected for modeling, and then the model was used to predict the two prediction sets to obtain the RPD, respectively. Second, the same operations such as 70% random sampling, modeling and prediction were repeated 50 times, and the mean and standard deviation of the RPD were calculated. The third step was to perform the same operations as PSSL on the CSSL and DSSL models. Finally, the one-way ANOVA was performed to check if the differences between the results obtained with the different scales SSLs were effectively significant.

## 3. Results and Discussion

### 3.1. Soil Spectrum

**Figure 3:**
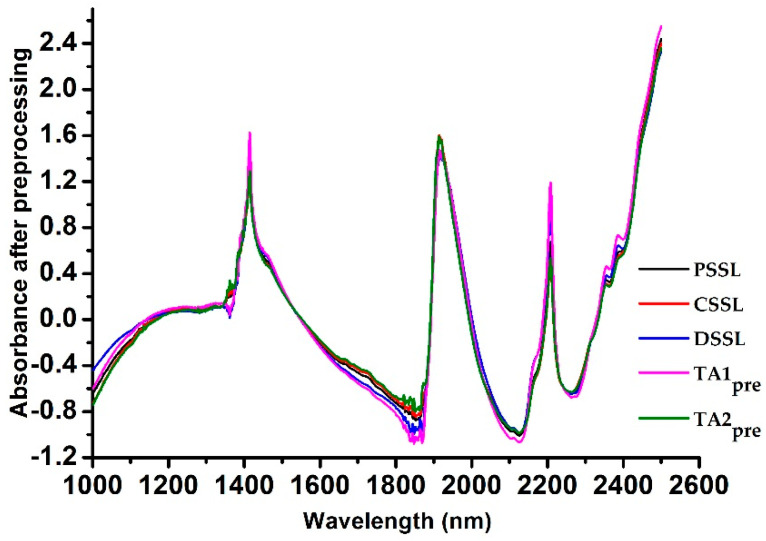
Average NIR spectra of soil sample sets after preprocessing.

Before establishing the calibration models, a preprocessing method of standard normal variate (SNV) combined with a detrending method with a polynomial order of 2 were used to eliminate the scattering effects in the spectra. The average NIR spectra after preprocessing of soil sample sets used in this work are shown in Figure 3.

### 3.2. ANOVA Test

Based on 70% samples randomly selected from the three SSLs′ sample sets, each model PSSL, CSSL, and DSSL was established by PLS. After 50 predictions were performed by each model on two prediction sets, the statistical results of the R^2^, RMSEP, and RPD are shown in Table 3.

Then, the one-way ANOVA test was performed using three scales of SSL as the single factor, and the results of the ANOVA to evaluate the effect of different scale SSLs on the RPD are shown in Table 4. The results show that different scales of SSLs have a significant impact on the predicted RPD (*p* < 0.001, Table 4), so the difference between the RPD obtained by different scales of SSL is valid.

### 3.3. Unspiked Models

Based on the three SSLs, each unspiked model (UM), PSSL-UM, CSSL-UM, and DSSL-UM, was established by PLS. Then, these models were used to predict the SOC contents of the samples in the two prediction sets, TA1_pre_ and TA2_pre_. The statistical results for the prediction performances of the UMs are shown in Table 5.

First, when the PSSL-UM established by the largest SSL was used to predict TA1_pre_ and TA2_pre_, the RPD was 1.90 and 2.17, respectively. This may be because the large-scale PSSL covered the geographic range and soil types of TA2_pre_, so a relatively good prediction effect was obtained. Meanwhile, when the soil type of TA1_pre_ was insufficient in the PSSL, the prediction effect was poor. These conclusions are similar to the results of Guerrero et al., indicating that large-scale soil libraries need to include the similar characteristics of soil samples in the target area to ensure a good prediction effect [25].

Second, the best prediction effect (RPD = 2.36) was obtained when using the CSSL-UM to predict TA2_pre_. Although the PSSL contained many more soil samples, CSSL may contain more samples similar to the soil characteristics in TA2_pre_ due to geographic location, so that the CSSL-UM achieved better prediction performance for TA2_pre_. Other researchers found similar results. Zeng et al. reported that samples used in the calibration model were geographically close to the target area, so the modeled soils were likely to have similar characteristics and spectra to the target area samples [26]. Furthermore, compared with a calibration model using all samples of a large-scale SSL, Liu et al. selected samples with similar spectral characteristics to the prediction set samples from the large-scale SSL for calibration modeling, and the ability to predict SOC content was significantly improved [27]. However, when the CSSL neither covered the geographical range of TA1_pre_ nor contained enough samples of the same soil type, the prediction effect of the CSSL-UM on TA1_pre_ was very poor (RPD = 1.46).

Third, when using the DSSL-UM established by the smallest SSL to predict TA1_pre_ and TA2_pre_, although the DSSL did not cover the geographic range of both target areas, the DSSL-UM had a moderate to good predictive ability (RPD = 2.05) for TA1_pre_ because the soil sample type in the DSSL was the same as TA1_pre_. Araújo et al. found similar results; when the types and spectra of soil samples in the calibration set and the target area were similar, quantitative analysis of the target area could be carried out even if it was based on a small SSL [28]. However, when some soil types in TA2_pre_ were missing in the DSSL, the prediction effect of the DSSL-UM on TA2_pre_ was poor (RPD = 1.83).

Comparing the performance of the UMs, we believe that it was a relatively stable method to use a large-scale SSL covering a wide geographic range and containing a full range of soil types to model, which can generally achieve better prediction results for target areas. However, among all the UMs, the CSSL-UM provided the best prediction results for TA2_pre_ (R^2^ = 0.81, RMSEP = 2.81, bias = 0.18, RPD = 2.36), indicating that the predictive ability of the model could be improved when the SSL contained more soil samples similar to the soil spectrum in target areas. Therefore, in order to obtain a better prediction effect, we studied whether the predictive ability of the model could be improved by means of spiking.

### 3.4. Spiked Models

The KS algorithm was used to select 5 or 10 spiking samples from each target area to establish the spiked calibration model. Then, based on different SSLs and numbers of spiking samples, each spiked model (SM), PSSL-SM, CSSL-SM, and DSSL-SM, was established by PLS, and these models were used to predict the SOC content of the samples in prediction sets TA1_pre_ and TA2_pre_. The statistical results of the prediction performances of the SMs are shown in Table 6.

**Figure 4:**
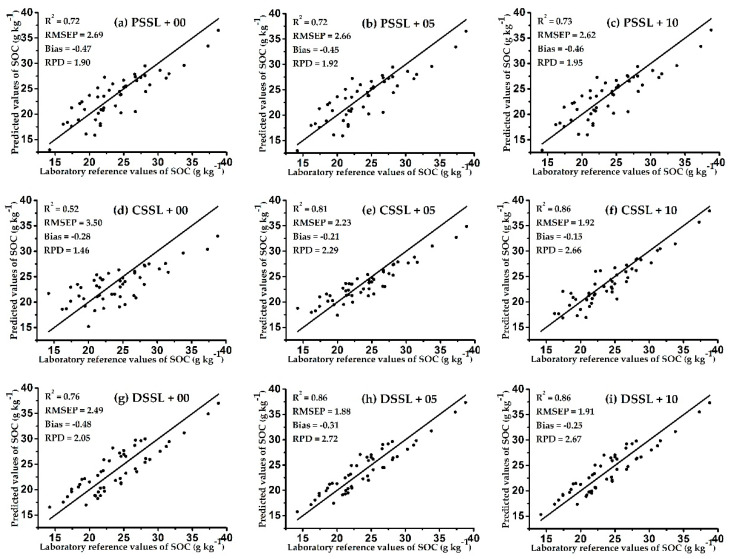
Scatter plots of predicted values and laboratory reference values of SOC of TA1_pre_ by each model. Scatter plots (**a**,**d**,**g**) show the prediction effect of the UMs; (**b**,**e**,**h**) show the SMs with 5 spiking samples from the target area; and (**c**,**f**,**i**) show the SMs with 10 spiking samples from the target area.

**Figure 5:**
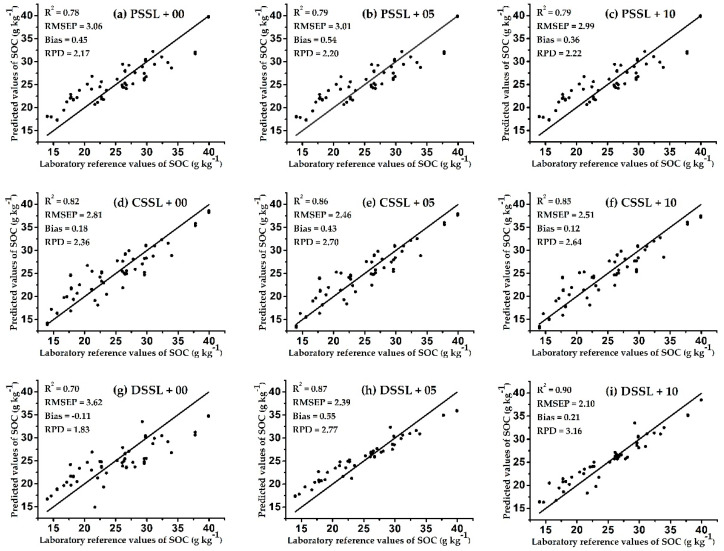
Scatter plots of predicted values and laboratory reference values of SOC of TA2_pre_ by each model. Scatter plots (**a**,**d**,**g**) show the prediction effect of the UMs; (**b**,**e**,**h**) show the SMs with 5 spiking samples from the target area; and (**c**,**f**,**i**) show the SMs with 10 spiking samples from the target area.

To formulate a more intuitive comparison before and after spiking, we needed to determine the differences between the prediction effects of spiking on the models established by the three SSLs containing different soil samples, soil types, and geographic ranges. Figure 4 and Figure 5 show scatter plots of the models’ predicted values for soil samples in prediction sets TA1_pre_ and TA2_pre_, and the corresponding SOC laboratory reference value for each sample. Each row in the figures represents a model based on the same SSL. The left, middle, and right-hand columns show the prediction effect of the UMs, the SMs with 5 spiking samples from the target area, and the SMs with 10 spiking samples from the target area, respectively. 

Three significant results are shown in Figure 4 and Figure 5. First, the prediction effect of each model was improved with the addition of spiking samples from the target areas, which indicates that the spiking method increased the predictive ability of the model. Other researchers also obtained similar results. Hong et al. found that adding spiking samples to the original model increased the accuracy of the prediction model [29]. In addition, compared with the UMs, improvements in the predictive ability of SMs was inversely proportional to the scale of the SSL. This may be because the smaller the original SSL, the higher the corresponding proportion of spiking samples in the SSL. Therefore, the established prediction model was more consistent with the soil characteristics of the target area, thus obviously improving its predictive ability. Guerrero et al. found a similar phenomenon by comparing eight SSLs of different sizes; the improvement effect of spiking was weaker in the large-scale SSL models, but more obvious in the small-scale SSL models [30].

Second, the method of spiking can solve the problem of poor prediction effect of the original model due to the lack of soil type samples in the target area. Comparing Figure 4d–f, it can be seen that when the CSSL contained a low proportion of soil types in TA1, after adding 5 and 10 spiking samples of the target area, the prediction effect of the CSSL models on TA1_pre_ was continuously improved (the RPD increased from 1.46 to 2.29 and 2.66). In addition, comparing Figure 5g–i shows that even when the DSSL lacked some soil type samples in TA2, after adding 5 and 10 spiking samples of the target area, the prediction effect of the DSSL models on TA2_pre_ was also continuously improved (the RPD increased from 1.83 to 2.77 and 3.16). However, when the original SSL already contained sufficient soil samples of similar characteristics to the target area, the predictive ability of the model was not significantly improved by spiking, and decreased due to saturation with the excessive number of spiking samples. For example, comparing Figure 4h,i shows that when 10 spiking samples were added, the predictive ability of the DSSL model on TA1_pre_ was reduced compared with 5 spiking samples (the RPD decreased from 2.72 to 2.67). In addition, the predictive ability of the CSSL model on TA2 _pre_ was also reduced when 10 spiking samples were added, compared with 5 spiking samples, as shown in Figure 5e,f (the RPD decreased from 2.70 to 2.64). Seidel et al. found a similar result; when the number of spiking samples reached 15–20, the predictive ability of the model declined due to saturation [31].

Third, the method of spiking caused the original SSL to not be affected by the geographic scope, so the model based on the small-scale SSL could be applied to the measurement of the target area outside the geographic scope of the SSL [32]. For example, the geographic scope of the DSSL did not include TA1 and TA2, but after adding 5 and 10 spiking samples, respectively, the corresponding prediction models achieved good results (RPD > 2.5), as shown in Figure 4h,i.

In my opinion, the number of spiking samples is firstly related to the method of selecting spiking samples. Guerrero et al. used the KS algorithm and selected 10% of the samples from the target area as the spiking samples according to the principle of uniform distribution of the principal component scores of the spectral data [30]. Seidel et al. used the KS algorithm and selected 12.5% of the samples from the target area as the spiking samples according to the principle of maximum differentiation based on the Mahalanobis distance of spectral data [31]. The results showed that these spiking samples had greatly improved the prediction accuracy of the model. Second, in order to make the spiking samples as representative of the soil characteristics of the target area as possible, the number of spiking samples should account for approximately 10% of the target set samples. In this work, for the small-scale SSL model DSSL-SM, the KS algorithm was used to select five spiking samples from two different target areas at a time (approximately 10% of the target set samples), the corresponding prediction models achieved good results (RPD > 2.5), as shown in Figure 4h and Figure 5h. Third, for the calibration model based on large-scale SSL, when the number of spiking samples is not enough to improve the prediction accuracy of the model, it is also considered to increase the weight of spiking samples to enhance their influence on the calibration model. Jiang et al. built the calibration model based on 608 soil samples, and selected 10 spiking samples from the target area. The results showed that the prediction accuracy of the model gradually improved as the weight of spiking samples was increased by 1, 5, and 10 times [33].

Overall, for TA1_pre_, the DSSL-SM with five spiking samples achieved the best prediction effect in all models, with an R^2^ of 0.86, RMSEP of 1.92, bias of −0.15, and RPD of 2.72, as shown in Figure 4h. For TA2_pre_, the DSSL-SM with 10 spiking samples achieved the best prediction effect in all models, with an R^2^ of 0.90, RMSEP of 2.10, bias of 0.21, and RPD of 3.16, as shown in Figure 5i. In comparison, Castaldi et al. used 12,128 samples from the large-scale LUCAS soil library to predict the SOC of three target sites in Belgium and Luxembourg, and the best prediction accuracy of the model was an RPD of 2.54 and R^2^ of 0.8 [34]. This indicates that the prediction performance of the model based on the small-scale SSL can also be compared with that of the large-scale SSL model after adding a few (5–10) spiking samples.

## 4. Conclusions

In this work, three SSLs of different scales (PSSL, CSSL, and DSSL) were constructed using NIR spectral data, and prediction models were established through the PLS algorithm to predict the SOC of two target areas. Since SOC is affected by regional climatic conditions, vegetation, land use, and other factors, the feasibility of the spiking method was further studied in order to improve the universality of the prediction model. For the new target area, it was expected that a model with high prediction accuracy could be built by using the existing SSL with the fewest spiking samples.

The results show that there was no correspondence between the model’s predictive ability and the scale of the SSL on which the model was based. In addition, the UMs (PSSL-UM, CSSL-UM, and DSSL-UM) established by the original SSLs were not accurate enough to predict the SOC of the new target areas. Then, the KS algorithm was used to select 5–10 spiking samples evenly distributed in the space defined by the first two principal components from the sample set of the target area. The results show that with the help of spiking samples, the predictive ability of each model was improved, and the improvement effect of the model based on the small-scale SSL was more obvious than that of the large-scale SSL. In this work, the DSSL-SM established by combining the original small-scale DSSL and a few spiking samples achieved good prediction of the SOC of two target areas. Therefore, it may not be necessary to build a large-scale SSL when using NIR spectroscopy to detect the SOC in a new target area. By building a small-scale SSL and measuring the chemical reference values of a few spiked samples, the SOC in a new target area can be accurately predicted.

## Figures and Tables

**Table 1:** Main characteristics of soil organic carbon (SOC) for all soil samples from the three soil libraries and two target areas.

Sample Set	Soil Type	Number	Range (g kg^−1^)	Mean (g kg^−1^)	SD (g kg^−1^)	Skew
PSSL	Red, yellow, paddy, and saline soil	714	6.6–46.3	24.9	7.28	0.14
CSSL	Red, yellow, and paddy soil	167	9.8–42.0	25.2	5.91	−0.20
DSSL	Paddy soil	102	7.5–40.0	22.2	6.07	0.50
TA1	Paddy soil	60	14.2–38.8	23.4	4.98	0.83
TA2	Red, yellow, and paddy soil	66	12.6–39.9	24.7	6.65	0.32

SD, standard deviation; PSSL, provincial soil sample library; CSSL, city soil sample library; DSSL, district soil sample library; TA, target area.

**Table 2:** Main characteristics of the two prediction sets.

Prediction Set	Soil Type	Number	Range (g kg^−1^)	Mean (g kg^−1^)	SD (g kg^−1^)	Skew
TA1_pre_	Paddy soil	50	14.2–38.8	24.0	5.11	0.74
TA2_pre_	Red, yellow, and paddy soil	56	14.0–39.9	24.7	6.63	0.37

**Table 3:** Statistical results of prediction performance of the different scale SSL models. RMSEP, root mean square error of prediction; RPD, residual predictive derivation; SD, standard deviation.

Model	TA1_pre_						TA2_pre_					
	R^2^		RMSEP (g kg^−1^)		RPD		R^2^		RMSEP (g kg^−1^)		RPD	
	Mean	SD	Mean (g kg^−1^)	SD (g kg^−1^)	Mean	SD	Mean	SD	Mean (g kg^−1^)	SD (g kg^−1^)	Mean	SD
PSSL	0.67	0.066	2.83	0.478	1.83	0.274	0.77	0.041	3.26	0.487	2.08	0.302
CSSL	0.51	0.048	3.52	0.504	1.45	0.198	0.81	0.032	2.88	0.301	2.32	0.233
DSSL	0.71	0.038	2.66	0.216	1.95	0.158	0.71	0.027	3.76	0.446	1.78	0.202

**Table 4:** Results of the ANOVA to evaluate the effect of different scale SSLs on the RPD.

Prediction Set	Source	Sum of Squares	Degrees of Freedom	Mean Square	*F*	*P*
TA1_pre_	SSL	5.333	2	2.666	57.41	0.000
	Error	6.828	147	0.046		
TA2_pre_	SSL	7.272	2	3.363	58.47	0.000
	Error	9.142	147	0.062		

**Table 5:** Statistical results of prediction performance of the unspiked models (UM). RMSEP, root mean square error of prediction; RPD, residual predictive derivation.

Model	TA1_pre_				TA2_pre_			
	R^2^	RMSEP (g kg^−1^)	Bias	RPD	R^2^	RMSEP (g kg^−1^)	Bias	RPD
PSSL-UM	0.72	2.69	−0.47	1.90	0.78	3.06	0.45	2.17
CSSL-UM	0.52	3.50	−0.28	1.46	0.82	2.81	0.18	2.36
DSSL-UM	0.76	2.49	−0.48	2.05	0.70	3.62	−0.11	1.83

**Table 6:** Statistical results of prediction performance of spiked models (SM).

Model	No.	TA1_pre_				TA2_pre_			
		R^2^	RMSEP (g kg^−1^)	Bias	RPD	R^2^	RMSEP (g kg^−1^)	Bias	RPD
PSSL-SM	5	0.72	2.66	−0.45	1.92	0.79	3.01	0.54	2.20
	10	0.73	2.62	−0.46	1.95	0.79	2.99	0.36	2.22
CSSL-SM	5	0.81	2.23	−0.21	2.29	0.86	2.46	0.43	2.70
	10	0.86	1.92	−0.15	2.66	0.85	2.51	0.12	2.64
DSSL-SM	5	0.86	1.88	−0.31	2.72	0.87	2.39	0.55	2.77
	10	0.86	1.91	−0.25	2.67	0.90	2.10	0.21	3.16

No., number of spiking samples.
